# Virus-like particles highly expressing DC-SIGN concentrate trimeric HIV-envelope proteins with noncovalently linked immunoreactive gp120 and gp41

**DOI:** 10.1186/1742-4690-9-S2-P359

**Published:** 2012-09-13

**Authors:** GN Vyas

**Affiliations:** 1 UCSF School of Medicine, San Francisco, CA, USA

## Background

We have hypothesized that membrane-bound macromolecular HIV-envelope proteins (mHIV-env) as a subunit, with gp120 and gp41 in their innate conformation, can be used as an effective immunogen to elicit broadly neutralizing antibodies (bnAb) against sexually or perinatally transmitted HIV-1. To test this hypothesis we have synthesized infection-free mHIV-env from the HIV-1 transmitted in humans (Vyas et al, Biologicals 2012;40:15-20). The process has been simplified for making large amounts of mHIV-env coupled with DC-SIGN expressing virus-like lipoparticles (VLP) as a candidate immunogen.

## Methods

Virus isolates of plasma-derived HIV-1 (PHIV) from infected blood donors while negative for anti-HIV (earliest acute infection) were selected for expansion in an optimized cell substrate (OCS) prepared from lymphocytes of four donors. Virions in the culture supernatants were inactivated by extracting membrane cholesterol with 200mM cyclodextrin for 4 hours to maximally expel p24, RT, and viral RNA from the permeabilized virions. The residual host/viral DNA/RNA in the inactivated virion shells were hydrolyzed with protease-free Benzonase without loss of gp120. The mHIV-env was coupled with VLP highly expressing DC-SIGN (Integral Molecular, Philadelphia, PA).

## Results

The inactivated virions apparently disintegrated into amorphous mHIV-env proteins when reacted with Benzonase. While the mHIV-env passed through membranes with 1,000kD cut off, it was retained by 30, 100, or 300kD cut off membranes as determined by gp120 EIA. The mHIV-env was coupled to DC-SIGN highly expressed on VLP. Analyses of DC-SIGN bound mHIV-env showed conformation-dependent immune reactivity with anti-gp120(b12). Remarkably, the gp41 noncovalently bound to gp120 was quantitatively detected by human monoclonal anti-gp41. The simplified method for preparing mHIV-env yielded trimeric heterodimers with an estimated molecular mass of 500kD, which was concentrated 10-100X with VLP expressing DC-SIGN.

## Conclusion

The VLP with mHIV-env bound to DC-SIGN at 10-100X more concentration than the 7-14 spikes per native virions provide a putative immunogen capable of inducing broadly neutralizing antibodies.

